# Differences in the Binding Affinity of an HIV-1 V2 Apex-Specific Antibody for the SIV_smm/mac_ Envelope Glycoprotein Uncouple Antibody-Dependent Cellular Cytotoxicity from Neutralization

**DOI:** 10.1128/mBio.01255-19

**Published:** 2019-07-02

**Authors:** Benjamin von Bredow, Raiees Andrabi, Michael Grunst, Andres G. Grandea, Khoa Le, Ge Song, Zachary T. Berndsen, Katelyn Porter, Jesper Pallesen, Andrew B. Ward, Dennis R. Burton, David T. Evans

**Affiliations:** a Department of Pathology and Laboratory Medicine, University of Wisconsin, Madison, Wisconsin, USA; b Department of Immunology and Microbiology, The Scripps Research Institute, La Jolla, California, USA; c International AIDS Vaccine Initiative Neutralizing Antibody Center, the Collaboration for AIDS Vaccine Discovery (CAVD) and Center for HIV/AIDS Vaccine Immunology-Immunogen Discovery (CHAVI-ID), The Scripps Research Institute, La Jolla, California, USA; d Department of Integrative Structural and Computational Biology, The Scripps Research Institute, La Jolla, California, USA; e Ragon Institute of MGH, MIT and Harvard, Boston, Massachusetts, USA; f Wisconsin National Primate Research Center, University of Wisconsin, Madison, Wisconsin, USA; Boston University; University of Washington

**Keywords:** ADCC, antibody function, human immunodeficiency virus, neutralizing antibodies, simian immunodeficiency virus

## Abstract

As a consequence of their independent evolutionary origins in apes and Old World monkeys, human immunodeficiency virus type 1 (HIV-1) and simian immunodeficiency viruses of the SIV_smm/mac_ lineage express phylogenetically and antigenically distinct envelope glycoproteins. Thus, HIV-1 Env-specific antibodies do not typically cross-react with the Env proteins of SIV_smm/mac_ isolates. Here we show that PGT145, a broadly neutralizing antibody to a quaternary epitope at the V2 apex of HIV-1 Env, directs the lysis of SIV_smm/mac_-infected cells by antibody-dependent cellular cytotoxicity (ADCC) but does not neutralize SIV_smm/mac_ infectivity. Amino acid substitutions in the V2 loop of SIV_mac_239 corresponding to the epitope for PGT145 in HIV-1 Env modulate sensitivity to this antibody. Whereas a substitution in a conserved N-linked glycosylation site (N171Q) eliminates sensitivity to ADCC, a lysine-to-serine substitution in this region (K180S) increases ADCC and renders the virus susceptible to neutralization. These differences in function correlate with an increase in the affinity of PGT145 binding to Env on the surface of virus-infected cells and to soluble Env trimers. To our knowledge, this represents the first instance of an HIV-1 Env-specific antibody that cross-reacts with SIV_smm/mac_ Env and illustrates how differences in antibody binding affinity for Env can differentiate sensitivity to ADCC from neutralization.

## Abstract (precis)

Here we show that PGT145, a potent broadly neutralizing antibody to HIV-1, directs the lysis of SIV-infected cells by antibody-dependent cellular cytotoxicity but does not neutralize SIV infectivity. This represents the first instance of cross-reactivity of an HIV-1 Env-specific antibody with SIV_smm/mac_ Env and reveals that antibody binding affinity can differentiate sensitivity to ADCC from neutralization.

## INTRODUCTION

HIV-1 and SIV_smm/mac_ evolved independently in apes and Old World monkeys and belong to phylogenetically distinct lineages of primate lentiviruses (1). Whereas HIV-1 is a result of the cross-species transmission of SIV_cpz_ from chimpanzees into humans (2, 3), SIV_mac_, which is widely used as a model for HIV-1 infection in nonhuman primates, is a result of the transmission of SIV_smm_ from sooty mangabeys to Asian species of macaques (4–6). As a reflection of their differing evolutionary histories, HIV-1 and SIV_smm/mac_ are antigenically distinct, and antibodies to the gene products of one virus typically do not cross-react with those of the other (7, 8). This is especially true for Env, which is rapidly evolving under the selective pressure of host antibody responses (9, 10). Thus, studies to assess protection by HIV-1-specific antibodies in nonhuman primates depend on the use of chimeric simian-human immunodeficiency viruses (SHIVs) expressing HIV-1 Env proteins (11).

Recent studies by our group and others have shown that the antibody-dependent cellular cytotoxicity (ADCC) of antibodies to the HIV-1 envelope glycoprotein generally correlates with their ability to neutralize viral infectivity (12–14). Nevertheless, instances of neutralization in the absence of ADCC were observed, revealing differences in Env epitopes exposed on the surface of virions versus infected cells (12). Instances of ADCC in the absence of neutralization were also observed for a few antibodies, but only against lab-adapted HIV-1_NL4-3_ (12). For primary HIV-1 isolates, every antibody with detectable ADCC activity neutralized viral infectivity, suggesting that the epitopes exposed on the surface of cells infected with these viruses are also accessible on functional Env trimers of virions (12).

In the present study, we show that PGT145, which recognizes a quaternary proteoglycan epitope at the V2 apex of HIV-1 gp120 (15–17), is able to opsonize SIV_smm/mac_-infected cells for NK cell lysis. The ADCC activity of PGT145 correlates with Env staining on SIV_smm/mac_-infected cells; however, this antibody does not neutralize SIV_smm/mac_ infectivity. To our knowledge, this represents the first instance of an HIV-1 Env-specific antibody cross-reacting with the Env proteins of SIV_smm/mac_ and reveals that differences in the affinity of antibody binding to Env can determine sensitivity to ADCC versus neutralization.

## RESULTS

### PGT145 targets HIV- and SIV-infected cells for antibody-dependent cellular cytotoxicity.

**FIG 1:**
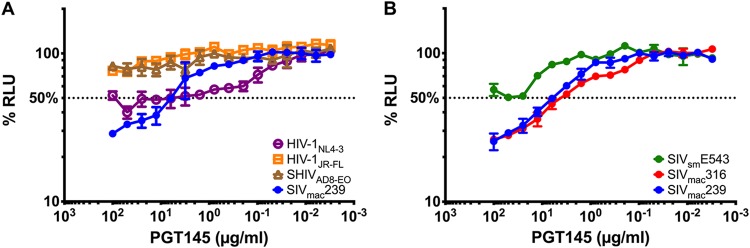
PGT145 mediates ADCC against HIV-, SHIV-, and SIV-infected cells. CEM.NKR-_CCR5_-sLTR-Luc cells infected with the indicated strains of HIV-1, SHIV, and SIV (A) and SIV alone (B) were incubated with an NK cell line (KHYG-1 cells) expressing human CD16 in the presence of the indicated concentrations of PGT145. ADCC activity was measured as the dose-dependent loss of luciferase activity in % RLU, compared to control wells containing no antibody and NK cells with either infected (maximal) or uninfected (background) target cells. The dotted line represents half-maximal lysis of infected cells, and the error bars represent the standard deviation of the mean from triplicate wells.

In the course of testing broadly neutralizing antibodies for ADCC activity against HIV-1-infected cells, we found that the V2 apex-specific antibody PGT145 mediated ADCC, not only against HIV-infected cells, but also against cells infected with SIV_mac_239 (Fig. 1A). To assess the breadth of this cross-reactivity, ADCC was measured against cells infected with three different SIV isolates, including SIV_mac_239, a neutralization-resistant virus that predominantly infects T cells (18, 19); SIV_mac_316, which is a macrophage-tropic isolate that is particularly sensitive to neutralizing antibodies (20, 21); and SIV_sm_E543-3, which is an independently derived SIV isolate that expresses a distinct, neutralization-resistant envelope glycoprotein (22). In addition to directing the killing of SIV_mac_239-infected cells, PGT145 mediated NK cell lysis of cells infected with SIV_mac_316 and SIV_sm_E543-3 (Fig. 1B), indicating that these responses to SIV-infected cells were not strain specific.

### PGT145 binds to cells infected with HIV, SHIV, and SIV.

**FIG 2:**
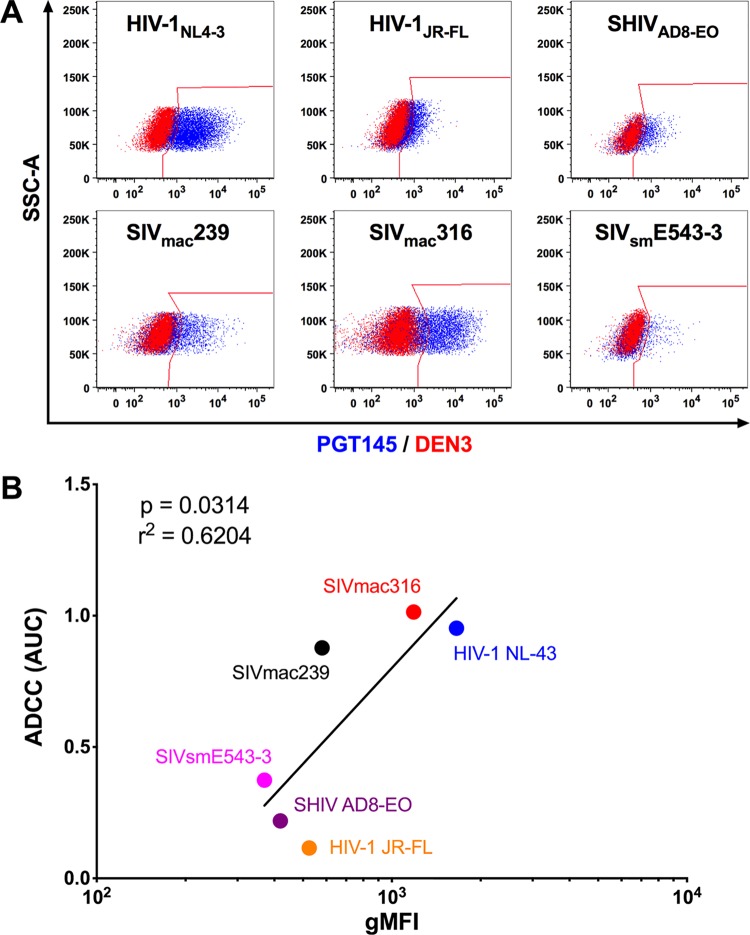
PGT145 stains cells infected with diverse lentiviral isolates. (A) Overlay plots show PGT145 (blue) versus DEN3 (red) staining of CEM.NKR-_CCR5_-sLTR-Luc cells infected with the indicated viruses. (B) Area-under-the-curve (AUC) values for ADCC responses were compared to the geometric mean fluorescence intensity (gMFI) of PGT145 staining on the surface of virus-infected cells by Pearson correlation. Virus-infected cells were identified by gating on the Gag^+^ CD4^low^ population, and PGT145 staining was detected with PE-conjugated anti-human IgG F(ab′)_2_.

To further investigate the unexpected lysis of SIV-infected cells by PGT145, binding of this antibody to HIV-1-, SHIV_AD8-EO_-, and SIV-infected cells was assessed by flow cytometry. Env staining on the surface of infected cells corresponded with susceptibility to ADCC for both HIV-1 and SIV envelope glycoproteins (Fig. 2A). In accordance with ADCC activity, PGT145 efficiently stained HIV-1_NL4-3_-infected cells and displayed low but detectable binding to HIV-1_JR-FL_- and SHIV_AD8-EO_-infected cells (Fig. 2A). PGT145 staining of SIV-infected cells was highest for cells infected with SIV_mac_316, followed by SIV_mac_239 and SIV_sm_E543-3 (Fig. 2A). Moreover, Env staining with PGT145 correlated with ADCC activity (*P* = 0.0314, Pearson correlation) (Fig. 2B).

**FIG 3:**
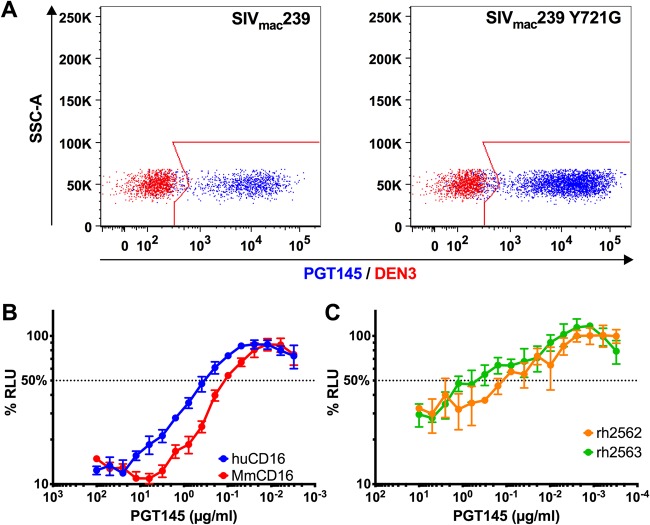
PGT145 binds to SIV-infected primary rhesus macaque CD4^+^ T cells and is recognized by rhesus macaque CD16. (A) Overlay plots show PGT145 (blue) versus DEN3 (red) staining of activated rhesus macaque CD4^+^ T cells infected with wild-type SIV_mac_239 or SIV_mac_239 Y721G. Virus-infected cells were identified by gating on the Gag^+^ CD4^low^ population, and PGT145 staining was detected with PE-conjugated anti-human IgG F(ab′)_2_. (B and C) ADCC responses were measured using an NK cell line expressing either human or rhesus macaque CD16 (B) or unstimulated PBMCs from two different macaques (C) by incubating SIV-infected CEM.NKR-_CCR5_-sLTR-Luc cells at a 10:1 effector-to-target-cell ratio in the presence of the indicated concentrations of PGT145. The dotted line indicates half-maximal killing, and the error bars represent standard deviation of the mean from triplicate wells.

Since species-specific differences in the glycosylation of Env may affect the binding of antibodies to glycan-dependent epitopes, we wanted to confirm that the staining of SIV-infected cells by PGT145 was not an artifact of SIV Env glycosylation in human cells. PGT145 was therefore tested for binding to SIV-infected rhesus macaque CD4^+^ lymphocytes. Activated PBMCs were infected with wild-type SIV_mac_239 or SIV_mac_239 Y721G, which contains a tyrosine-to-glycine substitution in a conserved endocytosis motif of the gp41 cytoplasmic domain that increases Env levels on the surface of infected cells (23, 24), and PGT145 staining was assessed by flow cytometry. In contrast to the control antibody (DEN3), PGT145 stained over 90% of the SIV-infected (CD4_low_ Gag^+^) cells (Fig. 3A). Consistent with Env-dependent binding, the fluorescence intensity of PGT145 staining was 1.6-fold higher on cells infected with SIV_mac_239 Y721G than on cells infected with wild-type SIV_mac_239 (Fig. 3A). PGT145 is therefore able to recognize SIV Env expressed on the surface of primary rhesus macaque lymphocytes in addition to human CD4^+^ T cell lines.

PGT145, which is a human IgG1 antibody, can also be recognized efficiently by rhesus macaque CD16. ADCC responses to SIV-infected cells were observed using NK cell lines expressing either human or rhesus macaque CD16 (Fig. 3B). Moreover, primary NK cells from two different rhesus macaques mediated similar ADCC responses in the presence of PGT145 (Fig. 3C). Thus, in addition to binding to Env on SIV-infected primary CD4^+^ T cells, PGT145 can interact with Fcγ receptors on the surface of macaque lymphocytes to mediate ADCC responses.

### PGT145 neutralizes HIV-1 and SHIV, but not SIV_smm/mac_.

**FIG 4:**
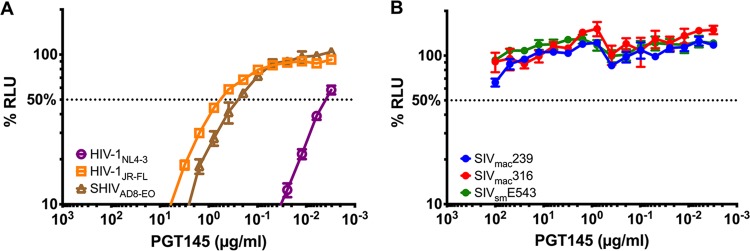
PGT145 neutralizes HIV-1 and SHIV but not SIV. The indicated viruses were incubated with serial dilutions of PGT145 for 1 h at 37°C before addition to TZM-bl cells (A) or C8166-SEAP cells (B). After 3 days, neutralization was measured as a decrease in luciferase (A) or secreted alkaline phosphatase (SEAP) (B) activity (RLU) compared to mock-infected or virus-infected control wells without antibody. The dotted line indicates 50% neutralization.

Since ADCC generally correlates with neutralization (12–14), we tested PGT145 for the ability to neutralize the same viruses that were susceptible to ADCC. As expected, PGT145 potently neutralized viruses expressing HIV-1 Env proteins, including HIV-1_NL4-3_, HIV-1_JR-FL_, and SHIV_AD8-EO_ (Fig. 4A). Corresponding to their sensitivity to ADCC, neutralization was most potent for HIV-1_NL4-3_ (IC_50_ = 0.004 μg/ml), followed by SHIV_AD8-EO_ (IC_50_ = 0.28 μg/ml) and HIV-1_JR-FL_ (IC_50_ = 0.62 μg/ml) (Fig. 4A). In contrast, despite being able to bind to Env on the surface of SIV-infected cells, PGT145 failed to neutralize any of the SIV isolates (Fig. 4B).

### PGT145 binds to a conserved conformational epitope at the V2 apex.

**FIG 5:**
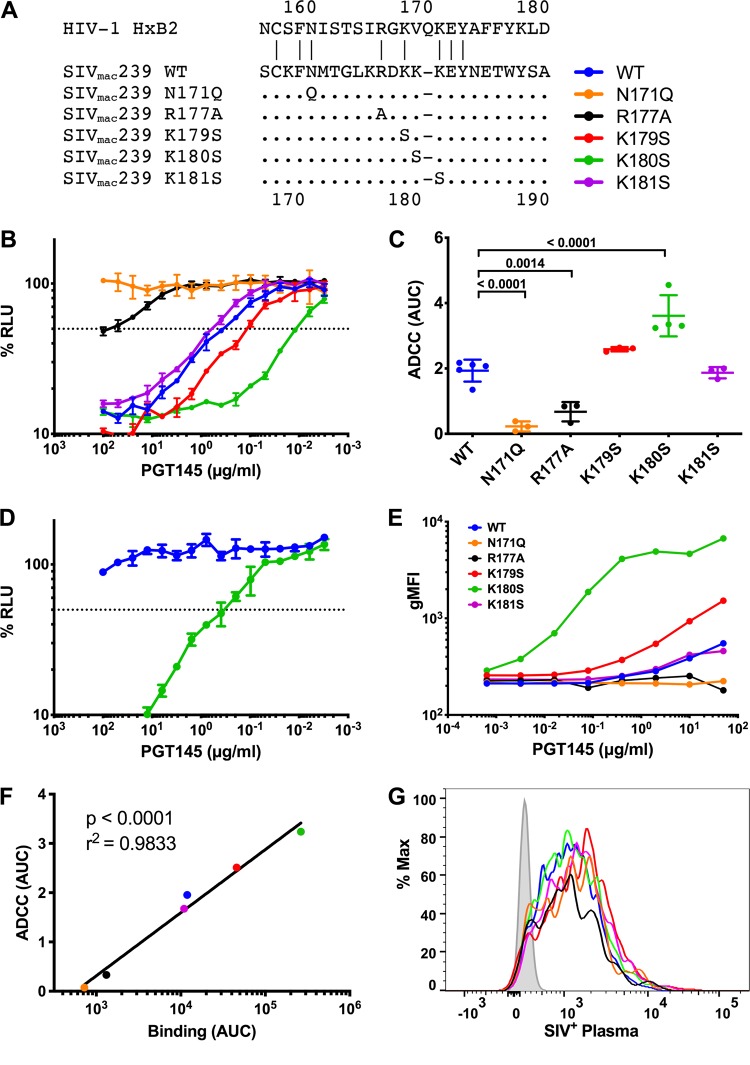
PGT145 binds to the V2 apex of SIV_mac_239 Env. (A) Amino acid substitutions were introduced in the V2 region of SIV_mac_239 Env. The amino acid sequence of HIV-1_HXB2_ is shown for reference, and the positions indicated in the legend correspond to SIV_mac_239 Env. Dots indicate identity, and dashes indicate gaps. (B) CEM.NKR-_CCR5_-sLTR-Luc cells were infected with SIV_mac_239 carrying the indicated Env mutations and incubated with an NK cell line expressing human CD16 in the presence of serial dilutions of PGT145. ADCC activity was measured as the dose-dependent loss of luciferase activity in % RLU. The dotted line represents half-maximal lysis of infected cells, and the error bars represent the standard deviation of the mean from triplicate wells. (C) Area-under-the-curve (AUC) values for ADCC were calculated and compared by one-way ANOVA with a Holm-Sidak adjustment for multiple comparisons. (D) Wild-type SIV_mac_239 and SIV_mac_239 K180S were incubated with serial dilutions of PGT145 for an hour before infecting C8166-SEAP cells. Neutralization was determined by calculating the loss of secreted alkaline phosphatase (SEAP) activity. The dotted line indicates 50% neutralization, and the error bars represent the standard deviation of the mean from triplicate wells. (E to G) CEM.NKR-_CCR5_-sLTR-Luc cells were infected with SIV_mac_239 V2 variants and stained with either PGT145 (E) or plasma from an SIV_mac_239-infected rhesus macaque (G). Fluorescence intensities are shown for viable infected (Gag^+^ CD4^low^) cells. PGT145 staining (AUC) was compared to ADCC (AUC) by Pearson correlation (F).

The binding of PGT145 to HIV-1 Env requires an N-linked glycan at position 160 and a patch of basic amino acids in the V2 apex of the envelope trimer (16, 17). To determine if PGT145 recognizes similar features of SIV_mac_239 Env, we introduced substitutions into the corresponding region of the SIV_mac_239 V2 loop and tested these mutants for susceptibility to ADCC (Fig. 5A). Elimination of a predicted glycosylation site at position 171 (N171Q), corresponding to N160 in HIV-1_HXB2_ Env, abolished the ability of PGT145 to mediate the lysis of infected cells (Fig. 5B and C), indicating that the recognition of SIV_mac_239 Env by this antibody is dependent on an N-linked glycan at this position. An arginine-to-alanine substitution at position 177 (R177A), corresponding to R166 in HIV-1_HXB2_, also greatly diminished the susceptibility of virus-infected cells to ADCC (Fig. 5B and C). Whereas lysine-to-serine substitutions at positions 179 and 181 had little effect (Fig. 5B and C), cells infected with a virus containing a substitution at position 180 (K180S) showed increased sensitivity to ADCC (Fig. 5B and C). Remarkably, this single amino acid change also rendered the virus susceptible to PGT145 neutralization (Fig. 5D).

Next, we investigated the relationship between ADCC and infected cell binding for the Env variants by flow cytometry. Whereas little or no staining was observed for cells infected with the N171Q and R177A variants at antibody concentrations as high as 50 μg/ml, PGT145 staining was detectable on cells infected with the K180S variant at 3.2 ng/ml, which was approximately 1,000-fold lower than the concentration necessary for a similar level of Env staining on cells infected with wild-type SIV_mac_239 (Fig. 5E). Thus, the avidity of PGT145 binding to Env on cells infected with the K180S variant is much higher than on cells infected with wild-type SIV. PGT145 binding to Env also correlated strongly with ADCC responses to the V2 variants (Fig. 5F) (*P* = 0.0001, Pearson correlation); however, staining with SIV^+^ plasma did not differ (Fig. 5G), indicating that differences in PGT145 binding reflect specific interactions with the V2 apex, rather than nonspecific differences in Env expression.

### PGT145 binding to SIV Env trimers.

**FIG 6:**
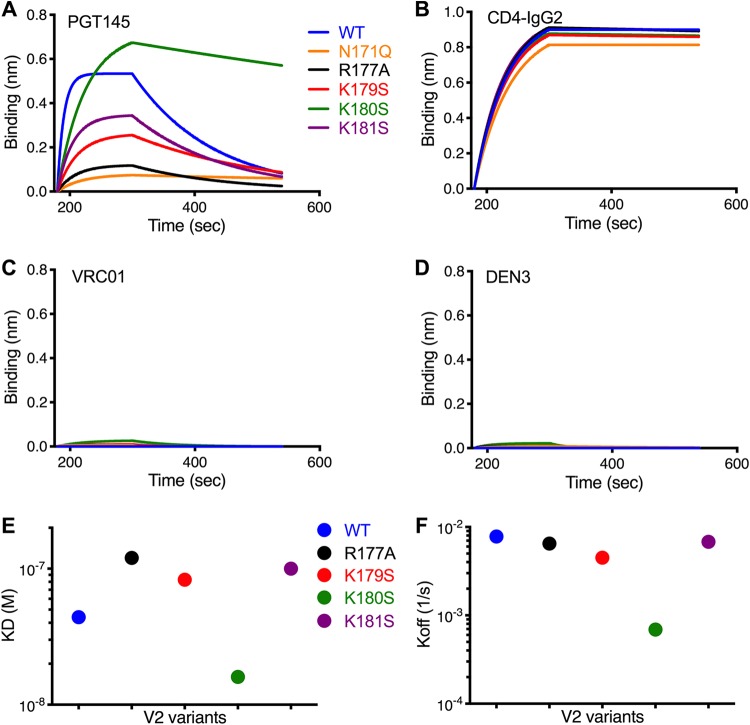
PGT145 binding to SIV Env trimers with V2 apex substitutions. The binding kinetics of PGT145 (A), CD4-IgG2 (B), VRC01 (C), and DEN3 (D) to soluble SIV_mac_239 SOSIP.664 Env trimers with the indicated substitutions in the V2 region was assessed by biolayer interferometry. Monoclonal antibodies immobilized on anti-human IgG-Fc-coated biosensors were dipped into trimer solutions (500 nM). The MAb-trimer interaction is represented as binding curves showing the rates of association (180 to 300 s) and dissociation (300 to 540 s). The dissociation constants (*K_d_*) (E) and dissociation rates (*K*_off_) (F) are plotted for PGT145 binding to each of the Env trimers.

Given the correlation between PGT145 binding to SIV-infected cells and ADCC, we hypothesized that differences in the susceptibility of the V2 variants to ADCC are a function of differences in the binding of PGT145 to Env trimers. To investigate this possibility, we generated soluble SIV_mac_239 Env SOSIP.664 trimers with substitutions in V2 that increase or decrease sensitivity to ADCC and analyzed their binding kinetics to PGT145 by biolayer interferometry (BLI). Whereas PGT145 binding varied (Fig. 6A), CD4-IgG2 bound to each of the SOSIP trimers with similar kinetics (Fig. 6B). As expected, binding was not detectable for the HIV-1 CD4 binding site antibody VRC01 or the dengue virus-specific control antibody DEN3 (Fig. 6C and D). Consistent with the specificity of PGT145 for Env trimers, cryo-electron microscopy 2-D class averages confirm that SIV Env affinity purified with PGT145 forms well-folded trimers (see Fig. S1 in the supplemental material). BLI analysis revealed that PGT145 bound to the wild-type Env trimer with a dissociation constant (*K_d_*) of 44 nM (Fig. 6A and E). Similar to the effects of the N171Q and R177A substitutions on PGT145 binding to infected cells and ADCC responses, these changes eliminated or dramatically reduced PGT145 binding to SOSIP trimers (Fig. 6A). Conversely, the K180S substitution increased the trimer binding affinity of PGT145 (Fig. 6A and E), primarily as a function of a lower off-rate (Fig. 6A and F). There were, however, some differences in PGT145 binding to recombinant Env trimers versus infected cells. Compared to infected cell binding, the range of PGT145 affinities for the SOSIP trimer variants was more compressed and the rank order exhibited some differences. The explanation for these differences is presently unclear but may reflect differences in the valency of PGT145 binding to Env on the surface of virus-infected cells versus recombinant SOSIP trimers or subtle differences in Env conformation and/or V2 glycosylation on infected cells compared to SOSIP trimers.

10.1128/mBio.01255-19.1 FIG S1 Cryo-electron microscopy of SIV_mac_239 Env trimers. SIV_mac_239 Env trimers were affinity purified using the V2 apex antibody PGT145 and analyzed via cryo-electron microscopy. A representative raw micrograph (scale = ∼100 nm) (A) and 2-dimensional class averages (B) indicate that SIV Env forms well-ordered, native-like trimers (scale bar = ∼4 nm). Download FIG S1, TIF file, 1.4 MB. Copyright © 2019 von Bredow et al. 2019 von Bredow et al. This content is distributed under the terms of the Creative Commons Attribution 4.0 International license.

Taken together, these results suggest that differences in the sensitivity of the SIV V2 variants to ADCC versus neutralization can be explained by differences in the affinity of PGT145 binding to Env trimers. Whereas the binding affinity of PGT145 to the wild-type SIV Env trimer appears to be sufficient to trigger ADCC responses by linking Env spikes on the surface of virus-infected cells to Fc receptors on NK cells, it is not of sufficient affinity for Env trimers on virions to permit neutralization of viral infectivity. In the case of the K180S variant, this single amino acid substitution raises the binding affinity by reducing the dissociation rate of PGT145-Env trimer complexes to an extent that SIV-infected cells become more sensitive to ADCC and cell-free virus becomes susceptible to neutralization.

## DISCUSSION

Owing to the independent evolutionary histories of HIV-1 and SIV_smm/mac_ and to the rapid evolution of their viral envelope glycoproteins in response to host immune responses, antibodies to the HIV-1 Env protein, including broadly neutralizing antibodies (bNAbs), typically do not cross-react with the Env proteins of SIV_smm/mac_. In the present study, we show that the HIV-1 Env-specific bNAb PGT145 can opsonize SIV-infected cells for elimination by ADCC but does not neutralize SIV infectivity. This cross-reactivity is not strain specific, since PGT145 mediated ADCC against cells infected with independent SIV isolates, nor is it a result of differences in Env glycosylation in human cells, since PGT145 also bound to Env expressed on the surface of SIV-infected primary rhesus macaque lymphocytes. The recognition of SIV Env by PGT145 was further verified by showing that substitutions in V2 corresponding to the HIV-1 V2 epitope for this antibody modulate the sensitivity of SIV-infected cells to ADCC.

A recent structure revealed that the CDRH3 loop of PGT145 forms a long *β*-hairpin that reaches down through the 3-fold axis of symmetry of the Env trimer to make contact with all three gp120 protomers (17). PGT145 binding to the Env trimer is monomeric and depends on asymmetric contacts with an N-linked glycan at position 160 (N160) and on electrostatic interactions between sulfotyrosine residues at the tip of the CDRH3 loop and basic residues of the V2 core (17). Although the precise molecular interactions between PGT145 and SIV Env are less clear, the dependence of PGT145 on SIV V2 residues N171 and R177, which correspond to HIV-1 V2 residues N160 and R166, respectively, indicates that PGT145 probably binds to SIV Env in a very similar fashion. In accordance with the contribution of residues N160 and R166 to the PGT145 epitope in HIV-1 Env, N171Q and R177A substitutions in the V2 loop of SIV dramatically reduce PGT145 binding and the sensitivity of SIV-infected cells to ADCC. Thus, PGT145 recognizes a conserved conformational epitope at the V2 apex of the SIV Env trimer.

The ability of PGT145 to mediate ADCC without neutralizing viral infectivity can be explained by its relatively low affinity for the SIV V2 apex. Sensitivity to ADCC strongly correlated with PGT145 binding to Env on the surface of SIV-infected cells, and although PGT145 did not block the infectivity of wild-type SIV, a single lysine-to-serine change at position 180 (K180S) was sufficient to render the virus susceptible to neutralization. Consistent with the effect of this change on increasing PGT145 binding to Env on infected cells, the K180S substitution increased the affinity of PGT145 for soluble SIV Env SOSIP.664 trimers as measured by biolayer interferometry. The higher affinity of PGT145 for the K180S trimer was primarily a function of a lower dissociation rate. These results suggest that the affinity of PGT145 binding to wild-type SIV Env, while sufficient to trigger ADCC by cross-linking multiple Env trimers on SIV-infected cells to Fc receptors on NK cells, is not high enough to occupy enough Env trimers on virions to block SIV infectivity. However, by stabilizing PGT145 binding to Env, the K180S substitution increases the affinity of PGT145 for Env on virions to a level sufficient to make the virus susceptible to neutralization.

PGT145 has a unique germ line configuration and an extended CDRH3 loop that distinguishes it from other V2 apex bNAbs (15, 16, 25). Accordingly, other V2 apex-specific bNAbs such as PG9 and PG16, which bind to similar quaternary epitopes consisting of an N-linked glycan and underlying basic residues, do not recognize SIV_smm/mac_-infected cells (12). Thus, the cross-reactivity of PGT145 with SIV_smm/mac_ Env appears to reflect unique structural features of this antibody. However, PGT145, as well as PG9 and PG16, is able to neutralize a subset of SIV_cpz_*Ptt* isolates (26). Although SIV_cpz_ is much more closely related to HIV-1 than SIV_smm/mac_, of the different classes of bNAbs that were tested, only 4E10 and 10E8, which target a well-conserved epitope at the membrane-proximal external region (MPER) of gp41, exhibited a similar breadth of neutralization (26). These observations therefore suggest a surprising degree of conservation of the V2 apex among phylogenetically diverse primate lentiviruses.

Conservation of the V2 apex may reflect important functional constraints on this region of Env. In order for Env to mediate fusion of the viral and cellular membranes, the trimer must transition from a closed to an open conformation in response to CD4 and coreceptor engagement that ultimately exposes the gp41 fusion peptide. Prior to CD4 binding, the Env trimer exists in a closed, metastable conformation held together largely by hydrophobic interactions (17). The proximity of positively charged residues at the core of the V2 apex as a result of the coalescence of basic residues from adjacent gp120 subunits may provide a destabilizing force that is necessary for the trimer to transition to an open conformation (17). Thus, while surface-exposed residues and the position of N-linked glycans may vary, the core of the V2 apex is more highly conserved to preserve key features essential for Env function. Consistent with this idea, structural data suggest that the breadth of HIV-1 neutralization by PGT145 is determined in part by general electrostatic interactions between anionic residues at the tip of the CDRH3 loop and an electropositive sink formed by basic residues at the core of the V2 apex, with relatively few contacts with specific residues (17).

Although instances of neutralization in the absence of ADCC have been observed, most antibodies that mediate ADCC against HIV-infected cells also neutralize viral infectivity (12–14). Thus, the ability of PGT145 to direct NK cell killing of SIV-infected cells without blocking SIV infectivity is unusual. In this case, the uncoupling of ADCC from neutralization can be explained by the relatively low affinity of PGT145 for the V2 apex of SIV Env, rather than differences in epitope exposure on the surface of infected cells versus virions. These findings illustrate how quantitative differences in antibody binding to Env can result in qualitative differences in antiviral activity with important implications for the selection of antibodies as immunotherapies to deplete HIV-1 reservoirs and for the development of antibody-based vaccines.

## MATERIALS AND METHODS

### Viruses.

Substitutions in SIV_mac_239 Env were introduced in the hemiviral plasmid p239SpE3′ by site-directed mutagenesis. SphI-PmlI fragments carrying the mutations were subcloned into the full-length infectious molecular clone SIV_mac_239 SpX. The infectious molecular clone for SIV_sm_E543-3 (22) was provided by Vanessa Hirsch, National Institute of Allergy and Infectious Diseases, National Institutes of Health.

### Virus production.

Virus stocks were produced by transfection of proviral DNA into HEK293T cells using GenJet transfection reagent (SignaGen). Culture supernatants were collected 48 and 72 h posttransfection, cell debris was removed by centrifugation, and aliquots of virus-containing supernatant were stored at −80°C. Virus concentrations were determined by anti-p27 or anti-p24 enzyme-linked immunosorbent assay (ABL, Inc.).

### ADCC assay.

ADCC activity was measured as previously described (27, 28). Target cells expressing luciferase (Luc) upon infection (CEM.NKR-_CCR5_-sLTR-Luc) were infected by spinoculation in the presence of 40 μg/ml Polybrene. At 4 days postinfection, cells were incubated with serial dilutions of PGT145 and an NK cell line (KHYG-1 cells) expressing either human or rhesus macaque CD16 at a 10:1 effector-to-target-cell ratio. For assays with primary cells, SIV-infected target cells were incubated with unstimulated rhesus macaque PBMCs. The dose-dependent loss of Luc activity after 8 h was measured as an indication of antibody-mediated killing of virus-infected cells. Infected target cells incubated with NK cells in the absence of antibody were used to measure maximal Luc activity, and uninfected target cells cultured with NK cells were used to determine background Luc activity.

### Neutralization assay.

Neutralization of viral infectivity was measured using reporter cell assays as previously described (9, 29, 30). Neutralization of SIV isolates was tested using C8166 cells expressing secreted alkaline phosphatase (SEAP) under the control of the SIV LTR (29). SIV_mac_239 (0.5 ng p27), SIV_mac_316_TM-open_ (15 ng p27), or SIV_sm_E543-3 (2 ng p27) was incubated with serial dilutions of antibody for 1 h at 37°C before adding 15,000 C8166-SEAP cells per well. After 3 days, SEAP activity was measured using a Phospha-Light SEAP detection kit (Applied Biosystems), and virus neutralization was calculated from reductions in RLU relative to cells incubated with virus but no antibody. Uninfected cells were measured to account for background SEAP activity. Neutralization of HIV-1 and SHIV isolates was similarly tested using a standard reporter assay (9, 30). After a 1-h incubation at 37°C with different concentrations of PGT145, 4 ng p24 (HIV-1_NL4-3_), 10 ng p24 (HIV-1_JR-FL_), or 20 ng p27 (SHIV_AD8-EO_) per well was used to infect TZM-bl luciferase reporter cells seeded at 5,000 cells per well the previous day. Neutralization was determined by measuring the loss of luciferase activity relative to cells infected in the absence of antibody, after deducting background luciferase activity from uninfected cells.

### Primary cells.

Peripheral blood mononuclear cells were isolated from whole blood using Ficoll-Pacque Plus (GE Healthcare). CD4^+^ T cells were subsequently enriched using the EasySep human CD4^+^ T cell isolation kit (StemCell Technologies) and activated using Dynabeads human T activator CD3/CD28 microbeads (Gibco). Activated CD4^+^ T cells were cultured in RPMI 1640 (HyClone) supplemented with 20% heat-inactivated FBS (HyClone), 2 mM l-glutamine (HyClone), 100 μg/ml Primocin (Invivogen), and 30 U/ml interleukin-2 (provided by Maurice Gately, Hoffmann-La Roche Inc., through the NIH AIDS Reagent Program). Infections were performed 3 days postactivation by spinoculation in the presence of 40 μg/ml Polybrene (Millipore).

### Flow cytometry.

Surface staining for Env was performed 3 days postinfection as previously described (23, 31). Antibody binding to Env was detected using a monoclonal antibody followed by a PE- or AF647-conjugated polyclonal anti-human IgG F(ab′)_2_. Cells were surface stained for CD45 (peridinin chlorophyll protein [PerCP]; clone 2D1) and CD4 (Alexa Fluor 700; clone RPA-T4) and then permeabilized and stained for intracellular Gag (fluorescein isothiocyanate [FITC]; clone 55-2F12). Nonviable cells were excluded using Live/Dead fixable dead cell aqua stain (Invitrogen), and data were collected using a SORP BD LSR-II flow cytometer (Becton Dickinson). After gating on viable CD45^+^ CD4^low^ Gag^+^ cells, the geometric mean fluorescence intensity (gMFI) of Env staining was calculated using FlowJo, version 9.7.7 (Tree Star, Inc.).

### Expression and purification of soluble SIV_mac_239 trimer.

SOSIP.664 Env trimer modifications were incorporated into the SIV_mac_239 Env coding sequence, as described previously (32). Amino acid substitutions were incorporated by using a QuikChange site-directed mutagenesis kit (Agilent Technologies, USA), according to the manufacturer’s instructions. All the mutations were confirmed by DNA sequence analysis (Eton Bioscience, San Diego, CA). Soluble recombinant Env trimers were expressed in HEK293F cells as described elsewhere (32). Briefly, plasmids encoding the SIV_mac_239 SOSIP.664 trimer and its V2 variants were cotransfected with a furin expression construct into HEK293F cells at a 3:1 ratio using PEI-MAX 4000 transfection reagent (Polysciences, Inc.). The secreted trimer proteins were purified from cell supernatants after 5 days using agarose-bound Galanthus nivalis lectin (GNL) (Vector Labs) columns as described previously (32). Affinity-purified proteins were separated by size exclusion chromatography using Superdex 200 10/300 GL columns (GE Healthcare) in phosphate-buffered saline (PBS). The purified trimers were stored at −80°C until use.

### Biolayer interferometry binding assay.

The binding kinetics of PGT145 and control MAbs with the affinity-purified trimers were analyzed by biolayer interferometry (BLI) using an Octet K2 system (FortéBio; Pall Life Sciences), as described previously (33). Briefly, the MAbs at concentrations of 10 μg/ml in PBS with 0.1% Tween (PBST) were immobilized onto anti-human IgG-Fc biosensors (AHC; FortéBio) for 60 s to achieve a binding response of at least 1.0 nm. After washing away the unbound MAb, the MAb-immobilized biosensor was dipped into a solution containing SOSIP.664 trimer protein (at a final concentration of 500 nM) as analyte and incubated for 120 s at 1,000 rpm. This was done by washing off the unbound trimer by placement into the PBST buffer for 240 s at 1,000 rpm. The 120-s and 240-s binding intervals denote the association and dissociation curves, respectively. The kinetic fits (1:1 binding kinetics model) were performed with the FortéBio Data Analysis version.9 software using the global fitting function.

### Cryo-electron microscopy.

SIV_mac_239 Env trimers were affinity purified with PGT145 and analyzed by cryo-electron microscopy. Three microliters of ∼6-mg/ml Env trimer was diluted 3:1 into 1.8 mm *n*-dodecyl-β-d-maltoside and plunge frozen on 1.2/1.3 C-Flat holey carbon grids (Protochips) with an FEI Vitrobot Mark IV (Thermo Fisher). Data were collected on an FEI Titan Krios instrument (Thermo Fisher) operating at 300 keV at a defocus range of –0.6 to −2.6 μm and controlled using Leginon (34). Movie micrographs were captured on a K2 Summit direct electron detector (Gatan) using an electron dose rate of ∼4.65 e^−^/pixel/s, a 250 ms^−1^ frame rate, and a total exposure time of 11.5 s, for a total dose of ∼50.4 e^−^/Å^2^. Frames were aligned and dose weighted with MotionCor2 (35), and the contrast-transfer functions were estimated with Gctf (36). The final magnified pixel size was 1.03 Å. Particles were picked and extracted with Relion2 automated particle picking using a Gaussian disc as a template (37). 2-D classification was performed using CryoSparc2 (38). The final set of 2-D classes shown in Fig. S1 in the supplemental material is composed of ∼62,000 particles.
