# Supplementary figures and images for: Differences in the Binding Affinity of an HIV-1 V2 Apex-Specific Antibody for the SIVsmm/mac Envelope Glycoprotein Uncouple Antibody-Dependent Cellular Cytotoxicity from Neutralization
Source: mBio. 2019 Jul 2;10(4):e01255-19. doi: 10.1128/mBio.01255-19 (PMC6606807; doi:10.1128/mBio.01255-19)

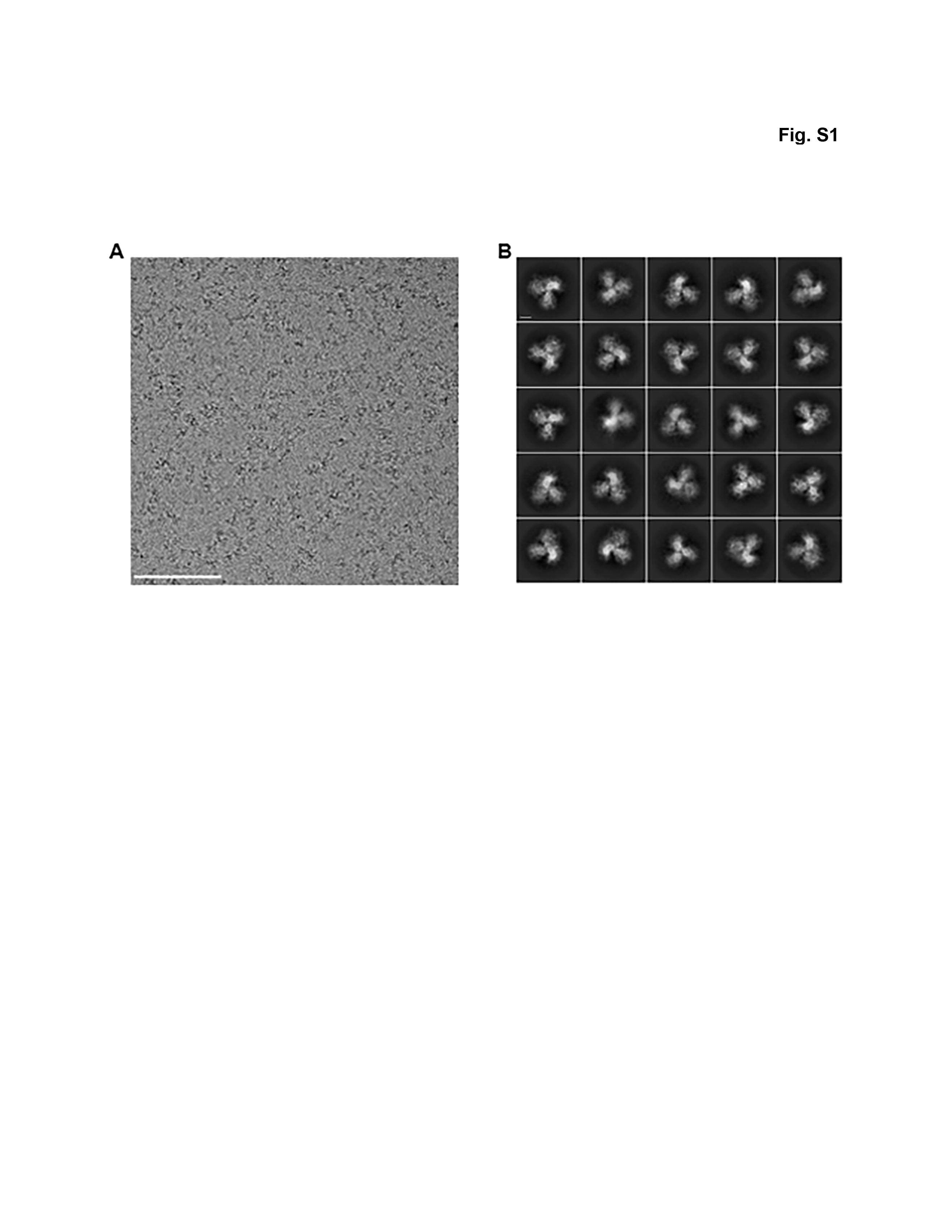

Supplement: FIG S1 [file mBio.01255-19-sf001.tif]
